# Exposure to Environmentally Relevant Concentrations of Genistein during Activation Does Not Affect Sperm Motility in the Fighting Fish *Betta splendens*

**DOI:** 10.1155/2014/865741

**Published:** 2014-01-02

**Authors:** Ethan D. Clotfelter, Hannah K. Gendelman

**Affiliations:** Department of Biology, Amherst College, Amherst, MA 01002, USA

## Abstract

Sperm collected from male fighting fish *Betta splendens* were activated in control water, water containing the ion-channel blocker gadolinium (a putative positive control), or water containing the isoflavone phytoestrogen genistein to determine the effects of acute genistein exposure on male reproductive function. Computer-assisted sperm analysis was used to quantify the proportion of sperm that were motile and the swimming velocity of those sperm. The highest concentration of gadolinium (100 **μ**M) tested was effective at reducing sperm motility and velocity, but neither concentration of genistein tested (3.7 nM or 3.7 **μ**M) significantly affected these sperm parameters. Our findings suggest that acute exposure to waterborne phytoestrogens during activation does not reduce the motility of fish sperm.

## 1. Introduction

Fish sperm are immotile in the gonads and are activated upon release into hypotonic (freshwater fishes) or hypertonic (saltwater fishes) solutions. Particularly among freshwater species, motility may last less than two minutes following activation [1, 2]. Lacking an acrosome, fish sperm must enter the egg mechanically by swimming through the micropyle. In many species, however, the brief duration of sperm motility is insufficient for the sperm to traverse more than half of the egg's circumference [3, 4]. Thus, sperm swimming velocity is one of the most important predictors of fertilization success in fishes with both internal and external fertilization [5–7].

Fish sperm have limited ability to adjust to physicochemical changes in their external environment [1, 8]. As a result, fish sperm are vulnerable to disturbance by numerous environmental contaminants. Direct exposure of sperm to contaminants, via activation of sperm in contaminated water, has been shown to reduce sperm motility, velocity, and fertilization ability [9, 10]. The mechanisms for such disruptions are largely unknown but may include damage to the sperm plasma membrane or axoneme, or consumption of adenosine triphosphate (ATP) [10]. In some cases, however, reductions in sperm motility and velocity are only observed at concentrations lethal to the fish themselves [10, 11].

Deficits in sperm motility due to environmental contamination can also occur indirectly when long-term exposure in maturing or adult fish perturbs sexual differentiation, gonadal development, or spermatogenesis. Lahnsteiner et al. [12] reported declines in trout sperm motility and swimming velocity when adult males were exposed to environmentally relevant levels of the endocrine disruptor bisphenol A. Similarly, Montgomery et al. [13] found that long-term exposure to the synthetic estrogen 17*α*-ethinylestradiol reduced sperm swimming velocity in the fighting fish, *Betta splendens*, most likely through a depletion of ATP reserves.

Phytoestrogens are a broad class of estrogenic compounds found in plants that have the potential to disrupt fish sexual development and gamete quality when they are concentrated and released into the environment due to human activity. Numerous studies have reported biologically active levels of phytoestrogens (particularly genistein, equol, and *β*-sitosterol) in effluent from wood pulp mills or sewage treatment plants or in agricultural soils [14–16]. At these concentrations, phytoestrogens can disrupt sexual differentiation and induce production of egg yolk protein in males [17–20]. The potential for fish populations to experience widespread and acute contamination from phytoestrogens is significant; undiluted plumes of pulp and paper mill effluent have been reported dozens of kilometers downstream from point sources [21].

Relatively little is known about the effects of phytoestrogen exposure on fish sperm quality. Sperm motility and concentration decreased in a dose-dependent manner in rainbow trout, *Oncorhynchus mykiss*, fed diets experimentally enriched with genistein, an isoflavone [18]. Stevenson et al. [22] exposed male fighting fish to waterborne genistein and *β*-sitosterol (a phytosterol) at environmentally relevant and pharmacological concentrations for four weeks but found that neither dose of either phytoestrogen had an effect on sperm quality. Sharpe et al. [23] found that *β*-sitosterol disrupted transcription of steroidogenic acute regulatory (StAR) protein in goldfish, *Carassius auratus*, which could interfere with steroidogenesis. Green and Kelly [24] incubated testes of channel catfish, *Ictalurus punctatus*, and walleye, *Sander vitreus*, in genistein and found a significant negative effect on sperm motility, ATP content, and *in vitro* fertilization rates in both species. The aforementioned studies all focus on phytoestrogen disruption of sperm maturation, but little work has been done on acute sperm exposure to phytoestrogens.

The purpose of the current study is to test the direct effects of environmentally relevant concentrations of genistein in activation water on the motility and velocity of sperm in the fighting fish, *Betta splendens*. By exposing sperm via activation water, the experimental design mimics the acute exposure that spawning fish might experience when phytoestrogen contamination is spatially or temporally variable. An ancillary goal of this research is to test the efficacy of gadolinium as a positive control in studies of fish sperm motility. Gadolinium blocks stretch-activated calcium ion channels, which are involved in sperm activation [25] and was deemed an appropriate positive control because genistein has been shown to affect motility of mammalian sperm by inhibiting spermatic calcium channels [26].

## 2. Materials and Methods

Sexually mature males from a domesticated strain of *Betta splendens* were purchased from a commercial supplier and acclimated for one week in the laboratory prior to each experiment. Fish were housed in individual, visually isolated 1-L beakers containing 800 mL of reverse-osmosis (RO) water reconstituted to a conductivity of 110–140 *μ*S using R/O Right (Kent Marine). Fish were fed freeze-dried chironomid larvae five times per week, water was maintained at 27°C, and the light cycle was kept at 14 : 10 L : D. Animal care protocols were approved by the Institutional Animal Care and Use Committee (IACUC) of Amherst College.

The first experiment was designed to test the effects of the ion channel blocker gadolinium on sperm activation and motility. Twenty-four *B. splendens* were anesthetized with buffered tricaine methanesulfonate (MS-222; Western Chemical) and sacrificed. The testes were removed and suspended in a quiescent state in 100 *μ*L of “catfish” sperm extender [27], which has been used successfully in a variety of species [3]. The sperm extender contained 5.52 g/L NaCl, 2 g/L KCl, 2.42 g/L Trizma HCl, and 3.75 g/L glycine, dissolved in distilled water at pH 7.5. The testes were then punctured 40 times with a needle to release the sperm. This method has been used successfully in several previous studies on sperm motility in *B. splendens* [13, 22].

Sperm samples were assigned to one of four treatments that differed in the composition of the activation water: control (no gadolinium), 25 *μ*M gadolinium, 50 *μ*M gadolinium, and 100 *μ*M gadolinium (*n* = 6 fish in each group). Gadolinium (Sigma-Aldrich Co.) was dissolved in trace quantities of ethanol; the control treatment contained only the ethanol vehicle. Four *μ*L of activation water (taken from aquaria) was mixed with 12 *μ*L of the sperm solution described above. Sperm were activated within 4 min after being extracted from testes. Five *μ*L of activated sperm sample was immediately mounted onto Leja 20-micron slides and viewed under a Nikon Eclipse E400 microscope. Using a SPOT Insight QE, Model 4.1 camera, three videos (approximately 6 sec in duration, 70 frames/sec) from different parts of each slide were recorded within 60 sec of sperm activation. The rapid assessment of motility ensured that our results were not affected by the relatively high osmolality [28]. Videos were analyzed with computer-assisted sperm analysis (CASA) using the Java plug-in for ImageJ [29]. Sperm number was counted in each slide view and standardized by multiplying the number of detected sperm by the dilution factor and dividing by the testes mass (g) of each fish. Parameters obtained for each fish were known indicators of fertilization success in a range of fish species [8]. These included percent motility, as well as the average curvilinear velocity (VCL; point-to-point velocity per sec), straight line velocity (VSL; velocity measured along a straight line from the first point of movement to the point furthest from the origin), and smooth path velocity (VAP; point-to-point velocity based upon the average path) for each video. For each sperm sample, the values of the above parameters were averaged for the three recorded video sequences to obtain a single parameter for each fish. The three velocity measures (VCL, VSL and VAP) were strongly positively correlated (*r* > 0.76, *P* < 0.001 for all correlations) and showed the same differences among treatment groups. Therefore, for simplicity we report results from VCL only.

To test the effects of genistein in activation water on sperm motility, we used 41 *B. splendens* from the same supplier and housed these fish in an identical manner. These fish were divided among four treatments: a negative control (no genistein in activation water), a positive control (100 *μ*M gadolinium), an environmentally relevant dose of 3.7 nM genistein, or a pharmacologic dose of 3.7 *μ*M genistein [14, 22, 30]. Each treatment group had 10 fish, except the 3.7 nM genistein group, which had 11 fish. Genistein (number G6649; Sigma-Aldrich Co.) and gadolinium were dissolved in ethanol prior to addition to activation water; the negative control treatment contained similar concentrations of ethanol alone. Testes were prepared and sperm motility recorded following the procedures described above.

SPSS version 15.0 was used for statistical analysis. Data were checked for normality prior to analysis and arcsin transformations were used when necessary. Differences were considered significant if *P* < 0.05 and means are presented ± SE. The statistical power of the analysis was estimated using the effects size conventions of 0.1, 0.25, and 0.40 for small, medium, and large effects, respectively, using G*Power version 3 [31].

## 3. Results

**Figure 1:**
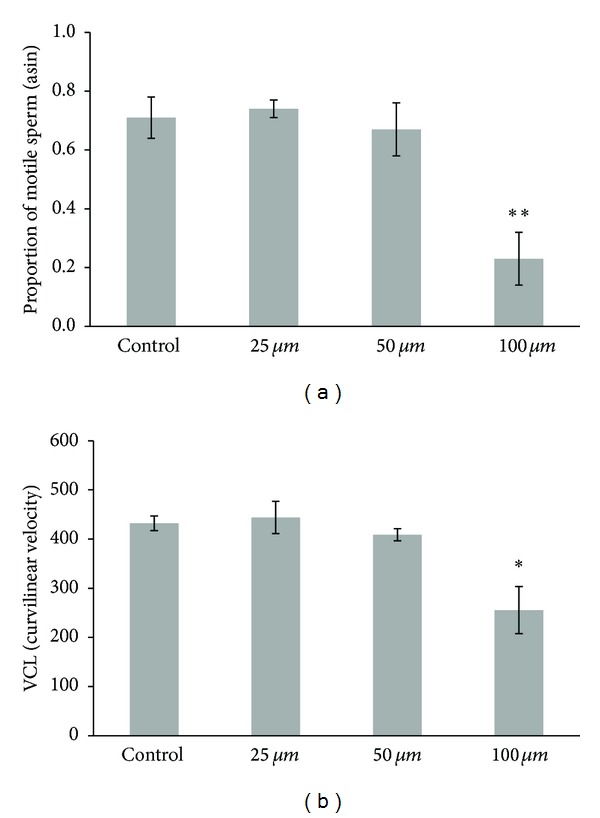
Motility (a) and curvilinear velocity or VCL (b) of *B. splendens* sperm exposed to negative control conditions or one of three doses of gadolinium (25, 50, or 100 *μ*M). Sperm motility data were arcsin transformed prior to analysis. *P = 0.001 and **P < 0.001. VSL and VAP (not shown here; see text for descriptions) were similar in statistical significance to VCL (*P* = 0.001 for both).

In the first experiment, activation water containing the highest dose of gadolinium (100 *μ*M) was found to have significant effects on *B. splendens* sperm. Gadolinium exposure resulted in fewer motile sperm (*F*_3,20_ = 12.77, *P* < 0.001; Figure 1(a)) and sperm with reduced curvilinear velocity or VCL (*F*_3,20_ = 8.18, *P* = 0.001; Figure 1(b)). Straight-line (VSL) and average path (VAP) velocity measures yielded similar results. A Scheffe's *post hoc* test revealed that motility and VCL were significantly reduced at 100 *μ*M compared to 0, 25, and 50 *μ*M (*P* < 0.018 for all). None of the other pairwise comparisons was significant (*P* > 0.88 for all). Thus, the 100 *μ*M dose of gadolinium was selected for use as our positive control in the subsequent experiment.

**Figure 2:**
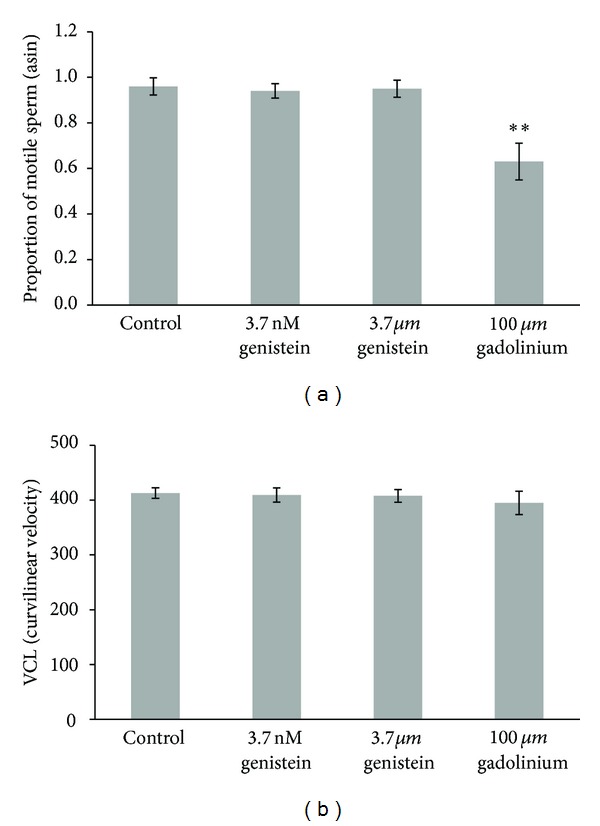
Motility (a) and curvilinear velocity or VCL (b) of *B. splendens* sperm exposed to negative control conditions, one of two doses of the phytoestrogen genistein (3.7 nM or 3.7 *μ*M), or the positive control gadolinium (100 *μ*M). Sperm motility data were arcsin transformed prior to analysis. **P < 0.001. Differences among treatments in VSL and VAP (not shown here; see text for descriptions) were similarly nonsignificant (*P* = 0.70 and 0.79, resp.).

In the second experiment, *B. splendens* sperm were activated in one of four treatments: negative control (ethanol vehicle only), positive control (100 *μ*M gadolinium), or one of two concentrations of genistein (3.7 nM or 3.7 *μ*M). Gadolinium exposure reduced the proportion of sperm that were motile (*F*_3,37_ = 9.1, *P* < 0.001; Figure 2(a)), but neither dose of genistein significantly affected the proportion of sperm that were motile (Figure 2(a)) or sperm VCL (Figure 2(b)) when compared to the negative control conditions (*P* > 0.78 for all Scheffe's *post hoc* comparisons). The statistical power of this analysis for small, medium, and large effect sizes was 0.07, 0.22, and 0.51, respectively.

## 4. Discussion and Conclusion

Fighting fish *B. splendens* sperm activated in water containing 100 *μ*M gadolinium were less likely to be motile and swam more slowly than sperm activated in control water or water with lower (25 or 50 *μ*M) concentrations of gadolinium. Sperm exposed to the highest dose of gadolinium showed similarly significant declines in all three measures of velocity (VCL, VSL, and VAP). This reduction in VCL, VSL, and VAP indicates asymmetric flagellar waveforms, perhaps due to the effect of gadolinium on intracellular Ca^2+^ concentrations [10].

Morisawa and Suzuki [2] were the first to demonstrate the particular role of potassium and calcium ions in the activation of fish sperm. Gadolinium ions (Gd^3+^) are known to block stretch-activated ion channels [32] and it has been proposed that their effect on intracellular Ca^2+^ concentration can initiate sperm motility [33]. Consistent with this, gadolinium reduced sperm motility in both puffer fish, *Takifugu niphobles*, and carp, *Cyprinus carpio*, sperm [34, 35]. These effects were reversible, suggesting that gadolinium blocked the stretch-activated channels, and were dependent on both dose and incubation time. The difference between our finding and those of Krasznai et al. [34, 35], namely, that they found significant effects of gadolinium at lower concentrations (e.g. 10 *μ*M), can be attributed to the longer incubation times used in their research. Thus, our gadolinium results are generally consistent with previous studies and support the conclusion that gadolinium is a suitable positive control for sperm motility studies in *B. splendens* and other fish species. Other potential positive controls include calcium ion chelating agent such as ethylene glycol tetra-acetic acid (EGTA) [25].

Activation water containing two environmentally relevant concentrations of the phytoestrogen genistein (3.7 nM and 3.7 *μ*M) caused no reduction in *B. splendens* sperm motility or velocity. Some studies report significant reductions in sperm motility and swimming velocity following phytoestrogen exposure in rainbow trout, channel catfish, and walleye [18, 24]. However, these studies employed different experimental approaches than the ones we used in the current study. Bennetau-Pelissero et al. [18] fed trout genistein-enriched diets for a full year prior to testing, and Green and Kelly [24] incubated catfish and walleye testes for 14 h in genistein solutions that were much more concentrated (up to 0.01 M) than the solutions we used. The genistein exposure that *B. splendens* sperm experienced in activation water may have been too brief to reduce ATP levels or increase oxidative damage, which are some of the proposed mechanisms by which phytoestrogens disrupt sperm motility in fishes [18, 24]. Future work should focus on the temporal changes in sperm motility following phytoestrogen exposure, particularly in fishes with long-lived sperm.

Our results were generally consistent with a previous study on the long-term effects of phytoestrogens on reproductive performance in this same species. Stevenson et al. [22] exposed adult male *B. splendens* to environmentally relevant levels of two waterborne phytoestrogens (genistein and *β*-sitosterol, and their mixture) for 4 weeks and found no detrimental effects on a battery of reproductive parameters including sperm motility and velocity, as well as sex steroid hormone levels, gonad size, and fertilization success. Thus, there was no evidence in that study that phytoestrogens accelerated spermatogenesis or disrupted steroid hormone production in *B. splendens* [22]. Coupled with those results, the current study suggests that acute exposure to environmentally relevant concentrations of genistein has few deleterious consequences for fish sperm. Future work could verify this conclusion by incubating testes in genistein solutions or by subjecting the sperm of species more sensitive than *B. splendens* to the acute effects of phytoestrogen exposure. Reproductive performance may still be compromised by phytoestrogens in aquaculture, however, where soy-based protein is a common ingredient in fish feed [18].
